# Probing the Optical Properties of Size-Selected Liquid-Phase Exfoliated γ-Indium Selenide

**DOI:** 10.3390/nano16150925

**Published:** 2026-07-27

**Authors:** Mikhail Kochiev, Muhammad Ahmad, Kevin R. Synnatschke, Sabrina Steffens, Tim Nowack, Zdenêk Sofer, Claudia Backes, Mohamed Benyoucef

**Affiliations:** 1 Institute of Nanostructure Technologies and Analytics (INA), Center for Interdisciplinary Nanostructure Science and Technology (CINSaT), University of Kassel, Heinrich-Plett-Str. 40, 34132 Kassel, Germany; 2 Institute of Chemistry, Center for Interdisciplinary Nanostructure Science and Technology (CINSaT), University of Kassel, Heinrich-Plett-Str. 40, 34132 Kassel, Germany; 3 Center for Advancing Electronics Dresden (CfAED), Faculty of Chemistry and Food Chemistry, Technische Universität Dresden, 01062 Dresden, Germany; 4 Institute of Physical Chemistry, Heidelberg University, Im Neuenheimer Feld 253, 69120 Heidelberg, Germany; 5 Department of Inorganic Chemistry, University of Chemistry and Technology Prague, Technicka 5, 166 28 Prague, Czech Republic

**Keywords:** liquid phase exfoliation, InSe, optical spectra

## Abstract

Van der Waals indium selenide is a promising material for next-generation optoelectronics due to its thickness-dependent band structure and high carrier mobility. Here, we investigate the optical properties of size-selected liquid-phase exfoliated γ-InSe nanosheets. The dispersions, composed of flakes with lateral dimensions below 100 nm, exhibit pronounced structural disorder and size-dependent optical behavior. Absorbance spectroscopy reveals systematic changes across size-selected fractions, enabling the extraction of quantitative metrics for estimating nanosheet lateral size and layers number. In addition, stability studies demonstrate significant degradation under ambient conditions, which is accelerated at elevated temperatures. Photoluminescence measurements on nanosheets exfoliated under inert conditions show broad, asymmetric emission with a clear blue-shift for smaller flakes, reflecting quantum confinement and dielectric screening effects. The emission characteristics further indicate a dominant contribution from localized states associated with disorder. These findings provide insight into the structure–property relationships in liquid-phase exfoliated γ-InSe and highlight its potential for solution-processed optoelectronic applications.

## 1. Introduction

γ-Indium selenide (γ-InSe) is a layered metal chalcogenide semiconductor that has attracted significant interest for optoelectronic applications owing to its high electron mobility and thickness-dependent electronic structure. The bandgap of 2D γ-InSe strongly depends on the number of layers, enabling broad spectral tunability over a broad spectral range [1]. Unlike transition metal dichalcogenides (TMDCs), monolayers and bilayers of γ-InSe possess an indirect bandgap, while thicker multilayers have a direct bandgap in good agreement with theoretical predictions [2]. This thickness dependence results in a broader range of optically active layer thicknesses than in TMDCs. The thickness of γ-InSe layers can be characterized by Raman spectroscopy [3,4,5,6,7], optical spectroscopy [5,8], and atomic force microscopy (AFM). To date, most reported optical and structural studies have focused on mechanically exfoliated nanosheets or epitaxially grown films. While the optical and structural properties of mechanically exfoliated γ-InSe have been extensively studied, considerably less attention has been paid to liquid-phase-exfoliated (LPE) γ-InSe. This study therefore focuses for the first time on the preparation of LPE γ-InSe with well-defined and quantified size and thickness and on investigating its optical properties both in dispersion and after deposition.

The carrier recombination rate in InSe nanoflakes increases as the layer thickness decreases [9]. For layer thicknesses below few tens of nanometers, carrier recombination is increasingly dominated by surface recombination, particularly in freshly exfoliated flakes. However, light absorption in InSe remains limited by the predominantly out-of-plane dipole nature of its electronic transitions. While the potential of γ-InSe for optical and photonic applications is high, several challenges remain regarding large-area sample preparation and material stability. It can be expected that γ-InSe samples produced by LPE may intrinsically overcome the limited light absorption of crystalline material due to their inherent structural disorder, which relaxes selection rules and enhances light–matter interaction.

Sonication-assisted LPE of InSe polymorphs [10,11] has been investigated in several studies. The choice of solvent remains under discussion, with *N*-methyl-2-pyrrolidone (NMP) identified as one of the most promising candidates [12,13]. Alternatively, 2-propanol [10] or water-ethanol mixtures [14] have also been employed for the exfoliation of β-InSe crystals. A typical limitation of sonication-assisted LPE is the simultaneous production of laterally small, thin nanosheets and larger, but thicker, flakes owing to the exfoliation mechanism [15]. To overcome this, alternative strategies such as electrochemical exfoliation [16] or megasonic exfoliation [17] have been suggested with the former having also been demonstrated for γ-InSe. However, such advanced strategies for exfoliation in liquids often require tedious optimization, are characterized by low throughput or introduce doping which still makes them a less-explored approach than sonication-assisted LPE. Furthermore, depending on the intended application, the production of laterally large and ultrathin nanosheets is not always required. Consequently, LPE remains a suitable preparation strategy, provided that the polydispersity in nanosheet size and thickness can be reduced. However, this aspect has not yet been investigated in detail.

The applicability of γ-InSe is further limited by its sensitivity to oxidation upon exposure to air and its degradation within a few days under ambient conditions. This degradation is further accelerated by elevated temperatures and laser irradiation [18,19,20]. To mitigate degradation, several protection techniques have been proposed [19,20,21], including encapsulation of InSe between hBN layers, controlled dry oxidation in pure O_2_, spin-coating with PMMA for short-term protection, and deposition of Al_2_O_3_ layers for long-term passivation. In this context, LPE offers several advantages, as sample exfoliation can be performed under inert conditions, e.g., in dried and degassed pyrrolidone-based solvents [22] or deoxygenated co-solvent systems [23].

In this study we address these points and first perform an in-depth investigation of LPE of γ-InSe including centrifugation-based size selection and the assessment of lateral size and thickness-dependent absorbance and extinction which allows us to establish quantitative metrics to assess average nanosheet dimensions from optical spectra. Furthermore, we determine the half-life of degradation for nanosheets of different sizes to clarify the role of defects (e.g., edges) and disorder for LPE nanosheets and investigate the photoluminescence response in nanosheet networks.

## 2. Materials and Methods

More details on initial sample preparation can be found in the Supporting Information (SI S1.1–S1.4).

### 2.1. Inert Exfoliation for Optical Studies

To prepare samples with minimal degradation, exfoliation was performed under inert conditions. Here, the exfoliation was done by probe sonication in a N_2_-filled glovebox using Sonopuls ultrasonic homogeniser HD 4100 with a TS 103 tip installed. In the first step, 480 mg of ground γ-InSe crystal was added to 80 mL of dried and degassed *N*-Methyl-2-pyrrolidone (anhydrous 99.5% NMP, Sigma Aldrich, Darmstadt, Germany) in a metal beaker and was sonicated for 1 h with an amplitude of 80%, and a pulse rate of 6 s on and 4 s off. The dispersion was split into three aliquots, each transferred to 50 mL centrifuge tubes in the glovebox, sealed with parafilm and then centrifuged at 6000 revolutions per minute (6 k rpm) for 2 h (outside the glovebox). After centrifugation, the vials were transferred back into the glovebox where sediment and supernatant were separated, followed by redispersion of the InSe sediment into fresh NMP for the second sonication for 7 h. The size selection was performed as before, but after each step, decantation was carried out in the glovebox. Fractions 100–300 *g* and 2–5 k *g* (where *g* is a free-fall acceleration) were subjected to optical characterization. The expression “2–5 k *g*” describes consecutive centrifugation steps. In this example, the sample was obtained by using a supernatant after centrifugation at 2 k *g* for a centrifugation run at 5 k *g* after which the sample is collected as sediment (for details see SI). For sample preparation, Si/SiO_2_ substrates were cleaned by bath sonication in acetone, isopropanol and deionized water respectively, for 10 min each. Films were prepared in the glovebox by drop-casting 25 μL of dispersion on the substrates and leaving the samples to dry in the glovebox.

### 2.2. Characterization

Atomic force microscope (AFM) measurements were carried out on a Dimension ICON3 scanning probe microscope (Bruker AXS S.A.S., Champs-sur-Marne, France) in ScanAsyst mode in air under ambient conditions using aluminum-coated silicon cantilevers (OLTESP-R3). The concentrated dispersions were diluted with solvent to optical densities < 0.1 across the resonant spectral region. Drops of the dilute dispersions (20 μL) were deposited on pre-heated (180 °C) Si/SiO_2_ wafers (0.5 × 0.5 cm^2^) with an oxide layer of 300 nm. After deposition, the wafers were rinsed with ~15 mL of water and ~15 mL of isopropanol and dried in a nitrogen flow. Typical image sizes were between 20 × 20 and 4 × 4 μm^2^ (depending on the nanomaterial size) at scan rates of 0.5 Hz with 1024 lines per image. Step height analysis was used to convert the apparent AFM thickness to a layer number as discussed in the manuscript. Previously published length corrections were used to correct lateral dimensions from cantilever broadening [24].

Optical extinction and absorbance measurements were carried out with an Agilent (Waldbronn, Germany) Cary 6000i spectrometer in quartz cuvettes. The spectrometer was equipped with an integrating sphere (external DRA-1800) for absorbance measurements. In this case, the cuvettes were placed in the center of the sphere. For this measurement, the optical density of the dispersions was adjusted to 0.3–0.4 at the peak. All spectra were acquired with 0.5 nm increments and 0.1 s integration time.

Photoluminescence (PL) spectra were measured using a µ-PL setup. The samples were placed under vacuum on a cold finger inside a helium-flow cryostat. The measurements were performed at a temperature of 10 K, unless otherwise specified. A continuous wave laser (λ = 532 nm) was focused by an objective with a numerical aperture of 0.7 to a spot of approximately 1 µm in diameter for optical excitation. The emitted light was collected through the same objective and is spectrally analyzed using a 0.75 m focal length spectrometer, equipped with a Peltier-cooled CCD detector. For time-resolved PL measurements, a 780 nm pulsed laser (∼50 ps) and a single photon avalanche photodiode mounted at the second spectrometer port were used. The overall temporal resolution was ∼0.3 ns.

## 3. Results and Discussion

For the production of solution-processable γ-InSe nanosheets by LPE, ground crystals were subjected to tip sonication in the organic solvent *N*-methyl-2-pyrrolidone. This solvent was chosen because it is commonly used for LPE and is known to produce colloidally stable dispersions for a range of materials [25,26]. Further, it has shown potential to protect nanosheets from oxidation under ambient conditions [27,28]. After probe sonication, the dispersion was size-selected using cascade centrifugation [29]. In this iterative centrifugation process, the dispersion is centrifuged in multiple steps with progressively increasing centrifugal acceleration (see SI). After each step, the nanosheets that settle in the sediment are redispersed and collected for analysis, while the remaining supernatant is centrifuged again at a higher speed in the subsequent step, thus producing size-selected fractions with decreasing size and thickness as the sample progresses through the cascade. The samples are denoted by the lower and upper centrifugation boundaries of the respective fraction.

### 3.1. Morphological Characterization

**Figure 1:**
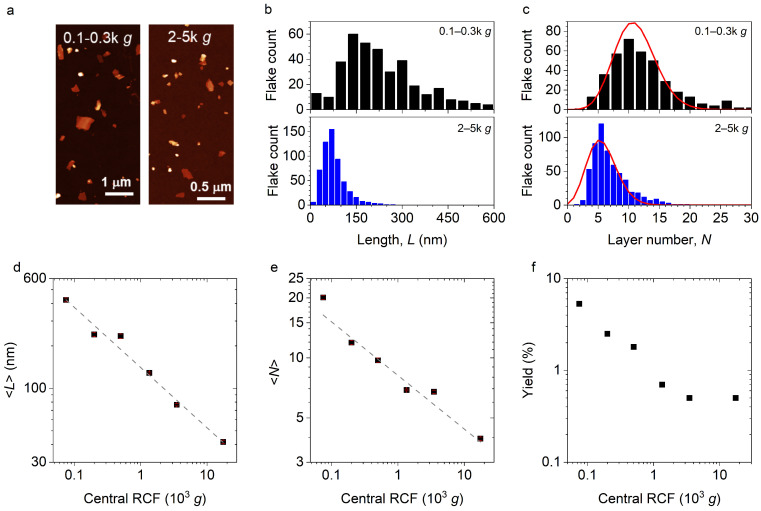
Characterization of nanosheet dimensions. (**a**) Representative AFM images of two size-selected fractions (100–300 *g* and 2–5 k *g*). (**b**) Histograms of the longest dimension (length *L*) and (**c**) layer number *N* of two fractions. Histograms for all fractions, see SI. Red curves are Poisson fits. (**d**) Average length <*L*> and (**e**) average layer number <*N*> plotted as a function of the midpoint of the set of relative centrifugal force (RCF) used for the size selection. Dashed lines in (**d**,**e**) are power law fits. (**f**) Plot of the yield as a function of the midpoint RCF.

The size-selected fractions were first characterized morphologically using atomic force microscopy (AFM) after drop-casting onto Si/SiO_2_ substrates. Representative images are displayed in Figure 1a for two fractions, i.e., 100–300 *g* and 2–5 k *g* (where *g* is a free-fall acceleration, see SI). AFM data for all fractions are shown in Figures S1–S3. To assess the size and thickness distribution, the longest dimension (length), dimension perpendicular to it (width) and the thickness of >300 individual nanosheets were measured for each fraction. The measured apparent AFM thickness was converted to a layer number using step height analysis as described elsewhere [30]. In brief, the heights of terraces of incompletely exfoliated nanosheets were measured and found to be multiples of a certain thickness which was attributed to the apparent height of one layer. For γ-InSe, we measured an apparent layer thickness of approximately 1.5 nm (Figure S4a). Accordingly, the measured AFM thickness was divided by 1.5 nm to obtain an estimate of the layer number. This allowed us to construct histograms of the length *L*, and the layer number *N*. Figure 1b displays representative *L* distribution histograms, while Figure 1c shows the corresponding *N* distribution histograms for the two fractions shown in Figure 1a. As expected, nanosheets isolated at higher centrifugation speeds are smaller and thinner and become less polydisperse. However, the lateral size and thickness distributions remain relatively broad under the chosen centrifugation conditions.

From the AFM data, the arithmetic average nanosheet dimensions were calculated for each fraction. The data is plotted as a function of the midpoint between the pair of centrifugal accelerations (expressed as relative centrifugal force in units of acceleration of the gravity *g*, RCF) in Figure 1d,e. A characteristic power law dependence is observed [31]. The average length <*L*> (Figure 1d) ranges from 40 to 450 nm with thicknesses <*N*> from 4 to 20 layers (Figure 1e). For additional size characteristics such as aspect ratios, see Figures S4b,c.

To further characterize the efficiency of the exfoliation, the yield of exfoliated nanosheets was determined for each fraction through filtration and weighing (see SI). The resultant data is plotted as a function of central RCF in Figure 1f. In the fraction of largest, thickest nanosheets, the yield is ~3% and drops to 0.3–0.4% for fractions containing the smallest and thinnest nanosheets. Photographs of the filtered films and scanning electron micrographs are displayed in Figure S5. They reveal disordered, porous networks of nanosheets characteristic for LPE samples.

### 3.2. Raman Spectroscopy

For further characterization, the filtered films were subjected to Raman spectroscopy (Figure S6). With 532 nm excitation (Figure S6c), all samples show the characteristic lattice vibrations of γ-InSe ($A_{1g}^{1}$ at ~115–116 cm^−1^, $E_{2g}^{1}$ at 177–178 cm^−1^ and $A_{1g}^{2}$ at 227–228 cm^−1^). Compared to the parent crystal (Figure S6a), no major changes are detected except for some broadening and reduced relative intensity of the $E_{2g}^{1}$ phonon. Since no such changes are observed when measuring the bulk crystal at higher laser power which results in visual degradation of the material, we attribute this difference to the disordered nature of the nanosheets in the LPE network rather than degradation. At 785 nm, the photoluminescence (PL) is discernible at 1000–3500 cm^−1^ (Figure S6b,d). After normalization to the $A_{1g}^{1}$ Raman mode, the relative PL intensity is clearly lower for the exfoliated nanosheets than for the parent crystal measured at low laser power which we attribute to disorder and edge effects. Furthermore, the PL exhibits a systematic blue-shift with decreasing layer number. This layer-number-dependent PL is discussed in more detail below. Overall, the Raman spectroscopy confirms that the exfoliated γ-InSe remains structurally largely intact with the primary difference being the additional disorder introduced by LPE.

### 3.3. Absorbance and Extincton Spectroscopy

**Figure 2:**
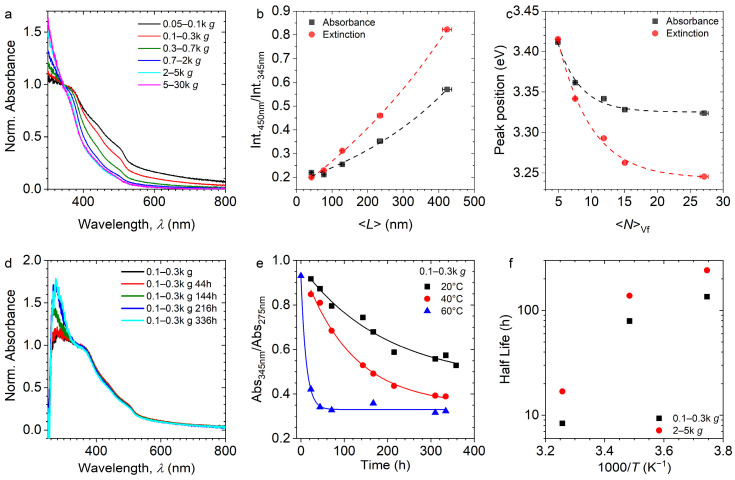
Optical extinction, absorbance and degradation. (**a**) Normalized absorbance spectra of size-selected dispersions measured in the center of an integrating sphere. For extinction spectra measured in transmission, see SI. (**b**) Plot of extinction and absorbance intensity ratio at 450 nm/345 nm as function of <*L*>. (**c**) Plot of extinction and absorbance peak position of the main transition determined from the second derivative as function of volume-fraction weighted average layer number <*N*>_Vf_. (**d**) Normalized absorbance measured after different time intervals after sample production of fraction 100–300 *g*. An increase in absorbance in the UV region is observed. (**e**) Decay kinetics after storage of the samples at different temperatures expressed as absorbance intensity ratio at 345 nm and 275 nm. Lines are exponential fits consistent with (pseudo) first-order decay to extract decay times. (**f**) Decay times as a function of inverse temperature for two samples 100–300 *g* and 2–5 k *g*.

Semiconducting 2D nanosheets such as transition metal dichalcogenides (TMDCs) [31] or transition metal thiophosphates such as NiPS_3_ [32] typically show a lateral-size- and thickness-dependent optical response in absorbance spectroscopy due to edge effects on the one hand and confinement and dielectric screening on the other. To test whether this is also the case for LPE γ-InSe, the size-selected dispersions were subjected to extinction and absorbance spectroscopy. The absorbance spectra were acquired at the center of an integrating sphere which collects scattered light (see Section 2.2). Normalized spectra are shown in Figure 2a. The absorption spectra consist of an intense band peaked at 300 nm with shoulders at about 375, 440 and 507 nm. The corresponding extinction spectra (recorded in transmission with scattering included) are shown in Figure S7. A dilution series shows good linearity of the optical response (Figure S8).

The absorbance spectra exhibit broad optical transitions which are most clearly resolved in the corresponding second-derivative spectra (Figure S7d). Overall, the spectral profile changes systematically across the size-selected fractions with smaller/thinner nanosheets exhibiting higher absorbance in the UV region and a reduced relative intensity of the transition at ~510 nm which is observed as a shoulder in the spectra but becomes hardly noticeable in the fraction containing the smallest and thinnest nanosheets.

Such changes in relative intensity ratios are typically attributed to edge effects [31]. To investigate their systematic scaling with lateral size, the intensity ratio of 450 nm/345 nm is plotted as a function of <*L*> for both absorbance and extinction spectra (Figure 2b). The intensity ratio is larger for the extinction spectra due to effects from nanosheet scattering which increases in relative intensity for larger/thicker nanosheets. In both cases, the data can be fit to an equation of the form $\left(y=\frac{Ax+B}{Cx+1}\right)$ consistent with edge effects [31] (dashed line). Based on this correlation, the average lateral size of LPE γ-InSe can be calculated for unknown samples using Equation (1). Because extinction measurements are experimentally more straightforward than absorbance measurements, the calibration below is given in terms of extinction:

(1) $$\left\langle L\right\rangle =\frac{0.149-\frac{{Ext}_{450nm}}{{Ext}_{345nm}}}{\left(-7.2\cdot 10^{-4}\right)\cdot \frac{{Ext}_{450nm}}{{Ext}_{345nm}}-0.001}.$$

Propagation of the nonlinear regression uncertainty indicates an uncertainty in the estimated lateral size of approximately ±7 nm for 20 nm nanosheets, increasing to ±45–50 nm for 500 nm nanosheets. Over most of the calibration range (50–500 nm), this corresponds to an approximately constant relative uncertainty of 8–10%. For more details on error estimation, see Supporting Information.

The thickness is typically reflected in excitonic shifts due to a combination of confinement effects and changes in dielectric screening with layer number. Empirically, exponential or logarithmic fits have been found suitable to describe the scaling of exciton energy with layer number as discussed elsewhere [33]. Peak positions were extracted from the second derivative of both extinction and absorbance spectra (SI Figure S7). The most prominent peak between 350 and 400 nm (3.1–3.5 eV) was chosen. Fitting the data yields a metric that enables the determination of the layer number of unknown samples according to Equation (2):

(2) $${\langle N\rangle }_{Vf}=-5.1\cdot ln\left(\frac{E_{ext}-3.243}{0.447}\right),$$

where ${\left\langle N\right\rangle }_{Vf}$ is the volume-fraction weighted layer number (which is larger than the arithmetic mean) and $E_{ext}$ is the energy (in eV) of the peak in the second derivative of the extinction spectra. Propagation of the nonlinear regression uncertainty indicates an uncertainty in the estimated layer number of approximately ±0.3 layers for monolayers, increasing to approximately ±1 layer for five-layer nanosheets and ±2–3 layers for 10-layer nanosheets. Owing to the asymptotic nature of the exponential calibration, the uncertainty increases rapidly for thicker nanosheets as the peak position approaches the limiting value $y_{0}$. For more details on error estimation, see the Supporting Information.

Independent replicate exfoliation and size-selection experiments (Figure S9) yielded the same dependence of the extinction ratio and peak position on centrifugal acceleration, demonstrating the reproducibility of the spectroscopic metrics and the robustness of the lateral size and layer-number calibrations.

### 3.4. Degradation

On the one hand, these metrics are of great practical use for quality control of the dispersions and for the rapid assessment of lateral size and thickness. On the other hand, understanding the optical extinction and absorbance spectra enables the use of optical spectroscopy to monitor potential degradation under ambient conditions or at elevated temperatures. To this end, two representative samples (100–300 *g* and 2–5 k *g*) were selected, and their absorbance was monitored at different time intervals for approximately two weeks. Samples were stored at room temperature or at 40 °C and 60 °C. The normalized absorbance spectra for one representative dataset (room temperature, 100–300 *g*) are displayed in Figure 2d; the complete dataset is shown in Figure S10. In all cases, the spectral shape changes with time, indicating degradation. In particular, the absorbance in the UV region increases relative to the absorbance in the visible range, suggesting the formation of oxides. Interestingly, the resulting spectral shape resembles that of laterally smaller nanosheets, suggesting that degradation may proceed from the edges towards the interior of the nanosheets.

To analyze the data, the intensity of the emerging peak at 275 nm in the normalized UV region was extracted and plotted as a function of time in Figure S11. The data clearly show that the degradation rate is greatly accelerated even by mild temperature increases to 40 °C or 60 °C. In all cases, the data is well described by an exponential decay consistent with (pseudo-)first-order reaction kinetics. From the fits, the decay time and the reacted fraction were extracted. At room temperature, the decay times are approximately 240 h for the smaller/thinner nanosheets and approximately 140 h for the larger/thicker nanosheets. These values are comparable to those reported for NiPS_3_ [33] and longer than those reported for black phosphorus [27] or TiS_2_ [28]. Somewhat counterintuitively, the smaller nanosheets degrade more slowly than the larger nanosheets. This observation suggests a complex degradation mechanism with kinetics extending beyond a simple first-order process. This interpretation is further supported by the observed deviation from Arrhenius behavior. According to the Arrhenius equation, the rate constant (or the inverse half-life for first-order kinetics) should scale exponentially with temperature according to the ratio of the activation energy and the thermal energy ($k_{B}T$). This relationship can be used to determine activation energies for the degradation of 2D nanosheets, as demonstrated for transition metal thiophosphates [33]. In the case of γ-InSe, however, the logarithm of the half-life does not scale linearly with the inverse temperature, preventing the determination of activation energies.

A simple degradation mechanism controlled solely by the accessible surface area would be expected to result in faster degradation of the smaller and thinner nanosheets because of their higher edge-to-area ratio. The opposite trend observed here therefore suggests that additional factors govern the degradation process. Possible contributions include differences in defect densities introduced during exfoliation, the relative abundance of edge and basal-plane sites, or size-dependent variations in surface chemistry and passivation by the surrounding solvent. The observed pseudo first-order kinetics should therefore be regarded as an effective description of the overall degradation process rather than evidence of a single elementary reaction step. Together with the deviation from Arrhenius behavior, these findings indicate that the degradation of liquid-phase exfoliated γ-InSe proceeds through multiple competing or consecutive processes with different temperature dependencies. Elucidating the individual reaction pathways, however, would require dedicated mechanistic studies that are beyond the scope of the present work.

An additional complication arises from the fact that the degradation is studied in liquid dispersion rather than in a homogeneous molecular system. In solution, the oxidation process is expected to proceed via encounters of the nanosheets with dissolved oxygen and/or water molecules within a solvent cage. Classical reaction kinetics distinguishes between diffusion-controlled and reaction-controlled regimes, where the observed macroscopic rate constant depends either on the diffusion-limited formation of the reactive encounter complex or on the subsequent chemical reaction within this complex. Consequently, the experimentally observed pseudo first-order rate constant may represent a convolution of diffusion, solvation, and chemical reaction steps rather than a single elementary process. This provides a plausible explanation for the observed deviation from simple Arrhenius behavior and further supports the notion that the degradation of liquid-phase exfoliated γ-InSe involves a more complex mechanism than a single thermally activated oxidation step. Disentangling these individual contributions would require dedicated kinetic studies, for example by extending the temperature range or varying the solvent properties (without compromising dispersion stability leading to aggregation).

Overall, the kinetics show that degradation occurs in ambient conditions, and also at room temperature. While the half-lives might be sufficient for further processing, we performed exfoliation under inert conditions for the remainder of the study to further suppress degradation. At the same time, the unexpected size dependence and deviation from Arrhenius behavior indicate that the degradation of liquid-phase exfoliated γ-InSe is governed by a more complex mechanism than a simple thermally activated oxidation process. Although elucidating the underlying pathways is beyond the scope of the present work, these findings motivate future mechanistic investigations of degradation chemistry of liquid-phase exfoliated γ-InSe.

### 3.5. Photoluminescence Properties

**Figure 3:**
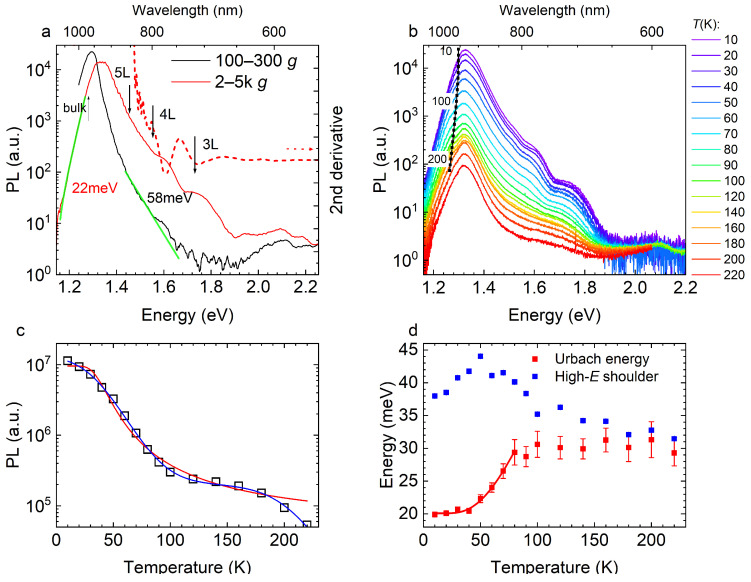
Photoluminescence properties. (**a**) PL spectra of the two InSe/Si samples: 100–300 *g* (black line) and 2–5 k *g* (red line). The red dashed line shows the second derivative of the short-wavelength emission of the 2–5 k *g* sample. Arrows show the PL maxima positions for the InSe nanosheets of different thickness (from [5]). Green lines—exponential fits for low- and high-energy shoulders of PL peak. (**b**) PL spectra of the 2–5 k *g* sample at the temperature range of 10–220 K. Black dots—bulk γ-InSe bandgap as reported in [30] and close to [33]. (**c**) Dependence of the integrated PL intensity (*I*_PL_) on the temperature *T* on a semi-log scale. Arrhenius fit and fit with model (4) are shown by red and blue curves, respectively. (**d**) Temperature dependencies of the Urbach energy (red symbols) and short-wavelength slope characteristic energy (blue symbols) of the PL spectra from (**b**). Red curve—the Einstein oscillator model fit with Equation (2).

To investigate the photoluminescence properties of LPE γ-InSe, we performed a comparative PL study of two representative samples. The PL spectra of the 100–300 *g* and 2–5 k *g* samples are shown in Figure 3a. The PL peak energies (i.e., optical band gaps) for the two samples are 1.29 eV (960 nm) and 1.33 eV (930 nm), respectively. Accordingly, the sample containing smaller nanosheets with a modal thickness of five layers (2–5 k *g*) exhibits a blue-shifted PL peak compared with the 100–300 *g* sample with a modal thickness of 10 layers. The PL peak position of the 100–300 *g* sample is close to that of the bulk γ-InSe A-exciton [5,8], whereas the PL peak of the 2–5 k *g* sample lies at a lower energy than that reported for mechanically exfoliated five-layer γ-InSe. The large Stokes shift observed for both samples reflect the fact that the strong absorption near 300 nm is attributed to transitions involving higher-lying electronic states, whereas the PL arises from radiative recombination of the localized A-exciton near the band edge. The residual Stokes shift relative to the A-exciton absorption is likely caused by carrier localization at defect- or surface-related states introduced during liquid-phase exfoliation, although a detailed investigation of these recombination pathways is beyond the scope of the present study. The emission linewidths of 53 meV for the 100–300 *g* and 83 meV for the 2–5 k *g* samples are attributed to the distribution of confinement energies arising from variations in nanosheet lateral size, thickness, as well as their surrounding dielectric environment.

When plotted on a logarithmic intensity scale, clear differences emerge on the short-wavelength side of the spectra. The 2–5 k *g* sample shows roughly one order of magnitude higher intensity in this region, indicating a higher concentration of thinner flakes, as expected from the centrifugation process (Figure 1c,e). Several sub-peaks are visible in the short-wavelength shoulder of the 2–5 k *g* sample. These features can be attributed to emission from flakes of varying thickness. To emphasize them, we calculated the second-derivative, *d*^2^*I*(*λ*)/*dλ*^2^, of the experimental spectrum after applying an appropriate smoothing procedure (red dashed line in Figure 3a). The minima of the second-derivative curve indicate the positions of unresolved emission components in the raw PL data. For example, the minimum at 1.73 eV corresponds to emission from a three-layer γ-InSe nanosheet at a low temperature [1,5]. It should be noted that the PL intensity does not directly represent the relative concentration of nanosheets with different thicknesses, since thicker nanosheets typically exhibit lower emission intensity. This behavior is consistent with previous PL studies on mechanically exfoliated γ-InSe nanosheets [34].

With increasing temperature to 220 K, the PL intensity is thermally quenched by about two orders of magnitude (Figure 3b). The modified Arrhenius formula

(3) $$I_{\mathrm{PL}}\left(T\right)=\frac{I_{\mathrm{PL}}\left(0\right)}{1+Ae^{\left(-E_{a}/k_{B}T\right)}},$$

provides a reasonable fit to the temperature dependence of integrated PL intensity (red line in Figure 3c), where *A* is the ratio of non-radiative to radiative recombination probabilities, *k*_B_ denotes the Boltzmann constant (0.086 meV/K), and *E*_a_ = 19 meV is the activation energy. This activation energy is comparable to the γ-InSe exciton binding energy (15–20 meV) [35,36,37,38,39]).

Alternatively, the temperature dependence of the integrated PL intensity can be fitted much better using an expression derived for highly disordered or amorphous semiconductors:

(4) $$I_{\mathrm{PL}}\left(T\right)=\frac{I_{0}}{1+Ae^{\left(T/T_{0}\right)}}.$$

The two-component expression in Equation (4) fits the data (blue line in Figure 3c) with two characteristic temperatures *T*_0_ = 15 K and *T*_1_ = 18 K (corresponding to 1.3 meV and 1.5 meV, respectively). These results further confirm that the radiative states are localized owing to the structural disorder in LPE γ-InSe. Moreover, the Arrhenius activation energy value is consistent with the Urbach energy *E*_struct_ (see below), supporting this interpretation.

The emission peaks are asymmetric (Figure 3b), with both the red and blue tails exhibiting near-exponential decay over certain spectral ranges. The exponential tail on the long-wavelength side is attributed to the Urbach tail in the density of states, reflecting structural disorder. The Urbach energy (Figure 3d) increases with temperature from 22 meV at a low temperature to 35–40 meV [39], consisting of a temperature-independent structural contribution and a temperature-dependent thermal contribution. The temperature dependence can be described by the Einstein-oscillator model:

(5) $$E_{U}\left(T\right)=\frac{A}{e^{\left(\Theta_{E}/T\right)}}+E_{U}^{\mathrm{struct}}$$

where $E_{U}^{\mathrm{struct}}$ is the temperature-independent structural disorder energy, Θ_E_ is the Einstein temperature, and *A* is a proportionality constant. A least-squares fit of the data in the range of 10–80 K yields *E*_struct_ = 20 meV, *A* = 36 meV and Θ_E_ = 200 K (equivalent to 17 meV).

The high-energy tail of the PL spectrum is often attributed to charge carriers with an effective temperature higher than that of the crystal lattice [40]. The characteristic decay energies obtained from exponential fits of the high- and low-energy tails of the PL spectra (Figure 3b) are summarized in Figure 3d. In our case, the short-wavelength decay energy does not vary monotonically with the lattice temperature. Furthermore, while exfoliated γ-InSe nanosheets exhibit a steeper high-energy tail at low temperatures [40,41], our LPE samples exhibit a characteristic decay energy of 35–45 meV, exceeding the Urbach energy. This is likely a consequence of the broad distribution of nanosheet sizes and thicknesses. The overlap of emission from nanosheets with different confinement energies broadens the high-energy side of the spectrum, resulting in a characteristic decay energy exceeding the intrinsic Urbach energy.

Several models have been proposed to describe the γ-InSe emission line shape. Venanzi et al. [40] employed a density-of-states approach combining the Urbach law for *E* < *E*_g_ and 3D density of states for *E* ≥ *E*_g_. At low temperatures, this model predicts a steep high-energy edge of the PL line, which depends on the temperature. Paylaga et al. used a carrier localization model [41] based on the product of the Voigt line shape and the complementary error function describing the distribution of potential fluctuations. This model can also reproduce asymmetric PL spectra in which the low-energy slope is lower than the high-energy slope. However, neither model adequately describes the PL spectra observed here.

However, the PL peak position shows very little temperature dependence (Figure 3b). The bulk γ-InSe bandgap temperature dependence is shown with black symbols for comparison (using the O’Donnell and Chen formula fit from [40], which is also close to the Varshni equation fit in [42]). This behavior suggests that emission is dominated by localized defect-related states [40,43,44,45].

## 4. Conclusions

In this work, we have systematically investigated the optical properties of size-selected liquid-phase exfoliated γ-InSe nanosheets. By combining cascade centrifugation with comprehensive structural and spectroscopic characterization, we established clear correlations between nanosheet dimensions and their optical response. Absorbance spectroscopy revealed size- and thickness-dependent spectral features, enabling the derivation of practical metrics for estimating lateral size and layer number in dispersions. Additionally, degradation studies highlighted the sensitivity of LPE γ-InSe to ambient conditions and elevated temperatures, underscoring the importance of inert processing for reliable optical characterization. Temperature-dependent photoluminescence measurements on drop-cast films demonstrated pronounced disorder-induced effects, including spectral broadening, asymmetric line shapes, and weak temperature dependence of the emission peak, indicating the dominance of localized states.

Overall, our results provide important insights into the interplay between confinement, disorder, and optical dynamics in liquid-phase exfoliated γ-InSe, and establish a foundation for its use in solution-processable optoelectronic and photonic applications.

## Data Availability

The data is available upon reasonable request.

## Supplementary Materials

The following supporting information can be downloaded at: https://www.mdpi.com/article/10.3390/nano16150925/s1, Materials and methods, additional figures of AFM and extinction/absorbance spectroscopy, uncertainty estimation of metrics.
