# Supplementary material for: Probing the Optical Properties of Size-Selected Liquid-Phase Exfoliated γ-Indium Selenide
Source: Nanomaterials (Basel). 2026 Jul 27;16(15):925. doi: 10.3390/nano16150925 (PMC13468010; doi:10.3390/nano16150925)
Supplement: Supplementary file 1 [file nanomaterials-16-00925-s001.zip › nanomaterials-4393413-supplementary.pdf]

Supplemental information

Probing the Optical Properties of Size-Selected Liquid-Phase Exfoliated γ-Indium Selenide

Mikhail Kochiev [1], Muhammad Ahmad [2], Kevin R. Synnatschke [3], Sabrina Steffens [4], Tim Nowack [2], Zdenêk Sofer [5], Claudia Backes [2,*] and Mohamed Benyoucef [1,*]

1 Institute of Nanostructure Technologies and Analytics (INA), Center for Interdisciplinary Nanostructure Science and Technology (CINSaT), University of Kassel, Heinrich-Plett-Str. 40, 34132 Kassel, Germany; kochiev.mikhail.ina@gmail.com

2 Institute of Chemistry, Center for Interdisciplinary Nanostructure Science and Technology (CINSaT), University of Kassel, Heinrich-Plett-Str. 40, 34132 Kassel, Germany; muhammad@uni-kassel.de (M.A.); tim.nowack@uni-kassel.de (T.N.)

3 Center for Advancing Electronics Dresden (CfAED), Faculty of Chemistry and Food Chemistry, Technische Universität Dresden, 01062 Dresden, Germany; kevin.synnatschke@tu-dresden.de

4 Institute of Physical Chemistry, Heidelberg University, Im Neuenheimer Feld 253, 69120 Heidelberg, Germany; s.steffens@fkf.mpg.de

5 Department of Inorganic Chemistry, University of Chemistry and Technology Prague, Technicka 5, 166 28 Prague, Czech Republic; zdenek.sofer@vscht.cz

* Correspondence: backes@uni-kassel.de (C.B.); m.benyoucef@physik.uni-kassel.de (M.B.)

1. Materials and Methods

1.1 Bulk material

γ-InSe crystal growth was performed from nonstoichiometric melt consisting of 52 at% In (5N; granules from Sigma-Aldrich) and 48 at% Se (5N; granules from Sigma-Aldrich). The precursors were placed in a quartz glass ampoule and evacuated to a base pressure of 7.5 Torr. The ampoule was melt-sealed with an oxygen–hydrogen welding torch, placed in a muffle furnace horizontally, and heated at 760 °C for 12 h. The melted reaction mixture was shaken several times to produce a homogeneous solution. Cooling was performed in a horizontal position to room temperature at a cooling rate 0.1 C/min.

1.2 Sonication-assisted LPE

For the initial exfoliation, the bulk crystals were ground with pistil and mortar and immersed in 80 mL *N*-methyl-2-pyrrolidone at a concentration of 20 g/L in a 100 mL metal beaker and subjected to a two-step sonication procedure with an external temperature of 5°C achieved through water cooling. A solid flathead tip was used (SonicsVXC-500) at 60% amplitude and a pulse sequence of 6 sec on and 4 sec off. The first sonication was performed for 1 h. The mixture including the solid residue at the bottom of the beaker was centrifuged in a Hettich Mikro 220R centrifuge equipped with a fixed-angle rotor 1016 at 2660 *g* for 1.5 h. The supernatant containing oxidized material and small nanosheets was discarded, and the sediment was collected in 80 mL of fresh solvent and subjected to a second sonication for 7h.

1.3 Size selection

Dispersions were size-selected by liquid cascade centrifugation in multiple steps of subsequently increasing centrifugation speeds. A Hettich Mikro 220R centrifuge equipped with a fixed-angle rotor (1195A) was used. Each centrifugation step was performed for 2 hours at 15°C. After each step, the sediment was redispersed for analysis in a reduced solvent volume (~1 mL) and the supernatant was used for the subsequent step. The following relative centrifugal force (speeds) were used: 50 *g* (~700 rpm); 100 g (~1k rpm); 300 g (~1.7k rpm); 700 g (~2.6k rpm); 2k *g* (~4.3k rpm); 5k *g* (~7k rpm); 30k *g* (~17k rpm). The expression “2-5k *g*” describes consecutive centrifugation steps. In this example, the sample was obtained

by using a supernatant after centrifugation at 2k *g* for a centrifugation run at 5k *g* after which the sample is collected as sediment.

1.4 Gravimetric filtering

The concentration of nanomaterial in different fraction was determined by filtering a known volume of dispersion though AlO_x membranes (pore size 20 nm) and washing with 1 L of isopropanol. Prior to weighing, membranes were dried overnight in vacuum at 60°C before and after deposition.

2. Figures

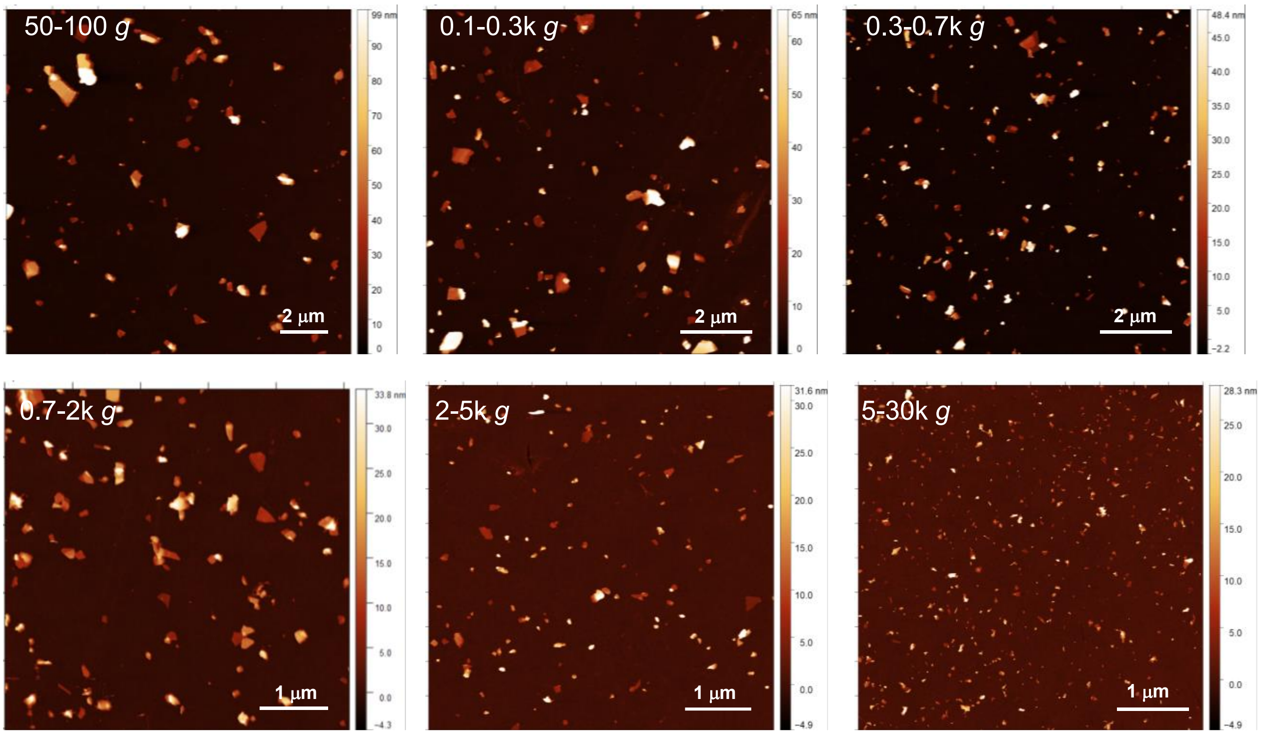

Figure S1: AFM images of the size-selected fractions.

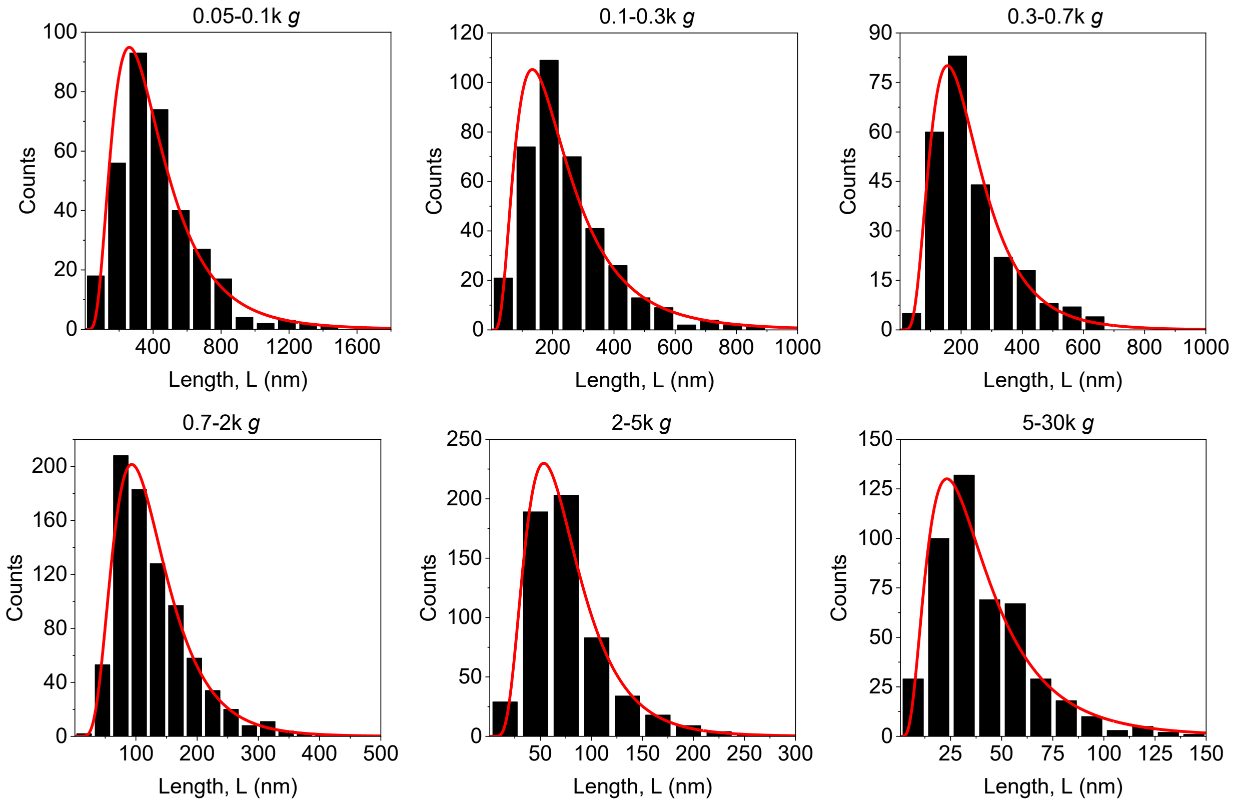

Figure S2: AFM length histograms of the size-selected fractions. Red curves – Poisson fits.

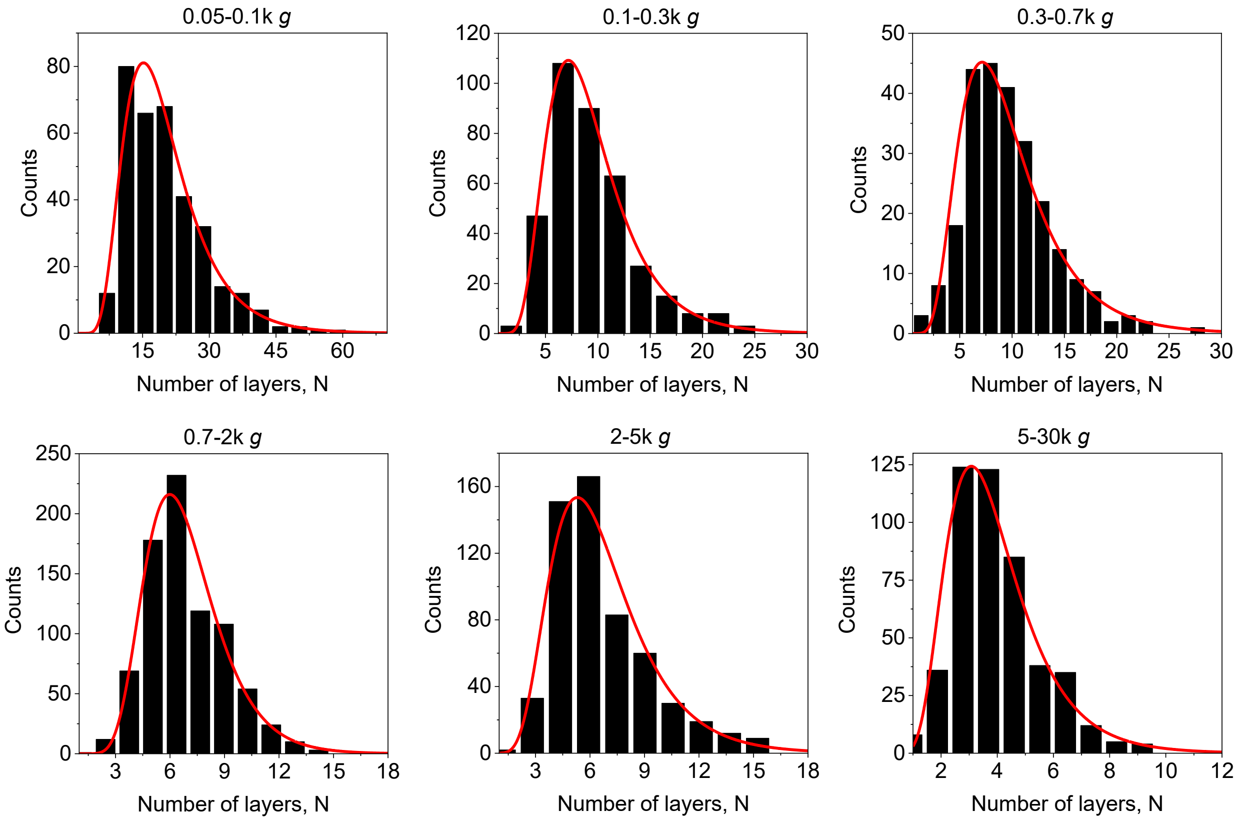

Figure S3: AFM layer number histograms of the size-selected fractions. Red curves – Poisson fits.

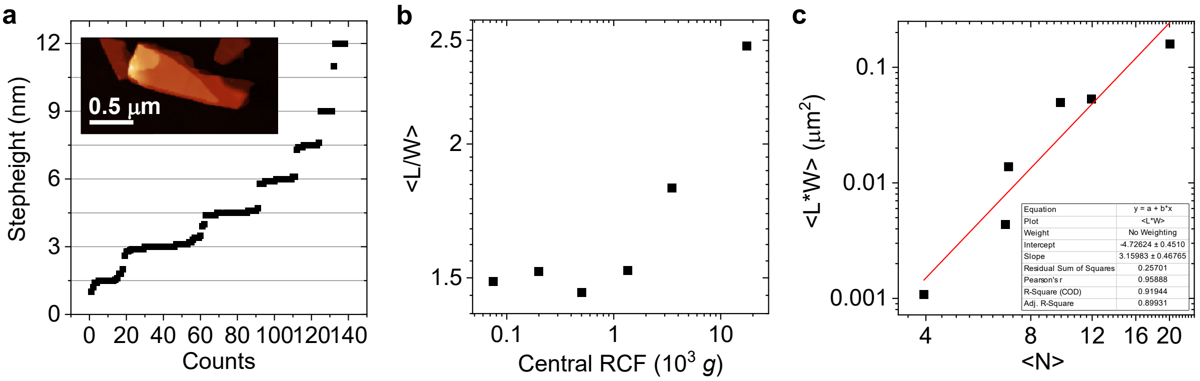

Figure S4: a) AFM step height analysis. The height of terraces on incompletely exfoliated nanosheets such as the one shown in the inset was measured and is plotted in ascending order. The step hight was found to be a multiple of 1.5 nm which is assigned to the apparent AFM height of one layer. Layer number is thus calculated by dividing the apparent AFM height of a nanosheet by 1.5 nm. b) Aspect ratio of length/width as function of midpoint of the set of centrifugal force used to produce a given fraction. c) Plot of average nanosheet area expressed as length multiplied by width as function of average layer number of the nanosheets in the size-selected fractions. A power law scaling is observed in agreement with equipartition of energy between exfoliation and tearing in sonication-assisted LPE.

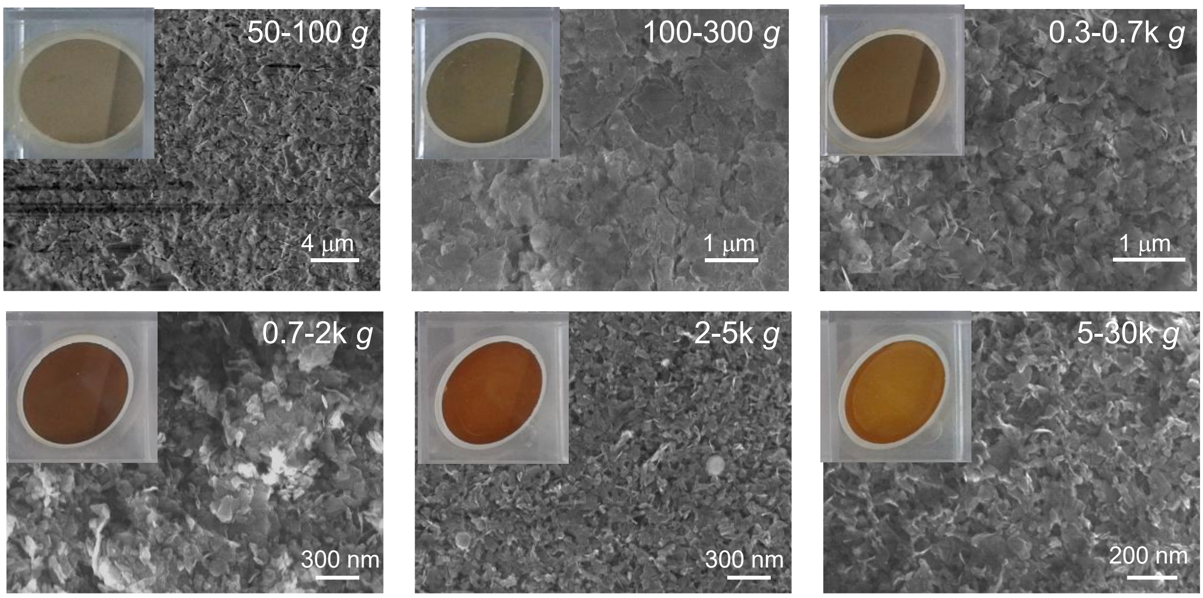

Figure S5: SEM images of filtered films of the size-selected dispersions. The inset shows photographs of the films demonstrating a change in colour with nanosheet size/thickness. SEM images were acquired with a JEOL JSM-7610F field emission scanning electron microscope (FE-SEM), using an In-lens Schottky field emission electron gun at 2.5×10^{-9} mbar. The images were measured with a dual (upper and lower) detector system consisting of collector-, scintillator-, light guide- and photomultiplier units for secondary electron imaging and an acceleration voltage of 15 kV.

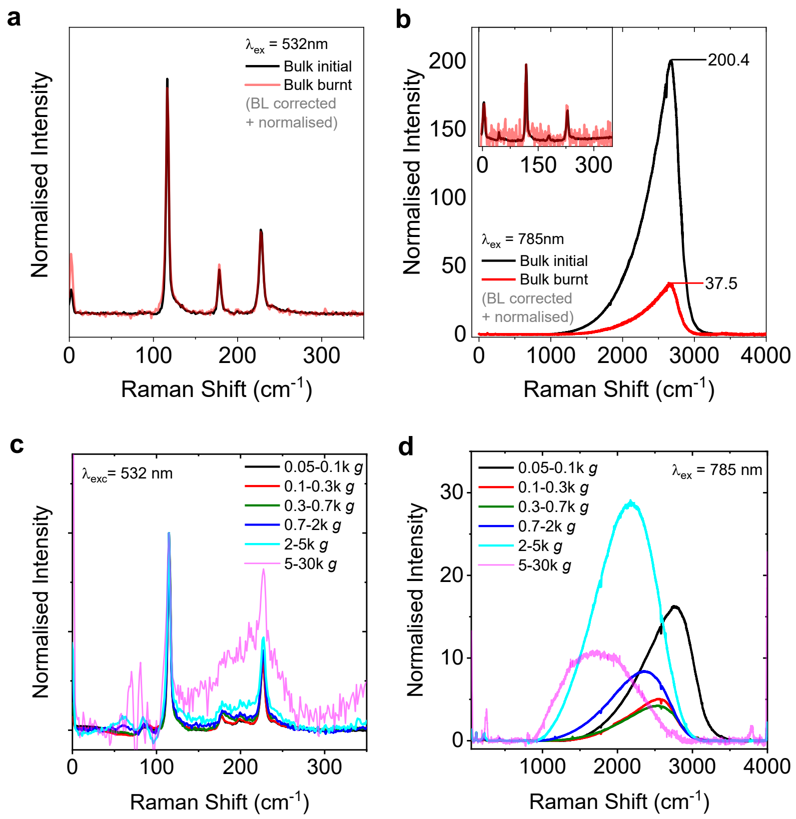

Figure S6: Raman characterisation of bulk crystal (a, b) and filtered films (c, d) using 532 nm (a, c) and 785 nm (b, d) excitation. The characteristic Raman modes of γ-InSe are clearly discerned and do not change significantly in the filtered films of the dispersion compared to the bulk except for some broadening. This is best observed with 532 nm excitation. The 785 nm excitation was used to measure the photoluminescence at 1000-3000 cm^{-1} Raman shift. After normalisation to the Raman modes of InSe, the relative intensity can be used as indicator of the emission strength. The PL/Raman ratio drops from ~200 in the bulk material to 5-30 in the exfoliated nanosheets. This is likely a result of edge effects, as edges represent defects. A blue-shift of PL position with decreasing thickness is observed. Raman measurements were carried out on a Renishaw InVia-Reflex confocal Raman microscope in air under ambient conditions. The Raman emission was collected by a 50× long working distance objective lens in streamline mode and dispersed by a 2400 l/mm grating with a maximal laser power of 1% (<10 μW). The spectrometer was calibrated to a silicon reference sample prior to the measurement to correct for the instrument response. Liquid dispersions were dropped (~20 μL) onto an aluminium support and left to dry in vacuum at 60°C overnight before measuring. For each sample, several spectra were accumulated on at least 3 different positions and averaged.

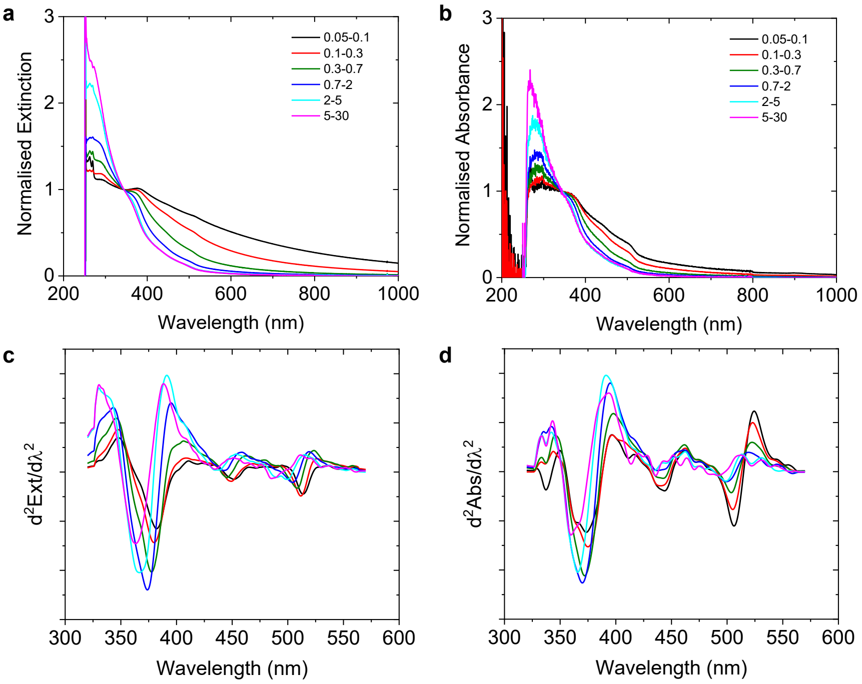

Figure S7: a) Normalised optical extinction and b) absorbance spectra of the size-selected dispersions. In panels c) and d), the second derivatives of the spectra are shown. Peaks are well resolved in the second derivative which allows to determine peak positions. These show a blue-shift with decreasing nanosheet thickness.

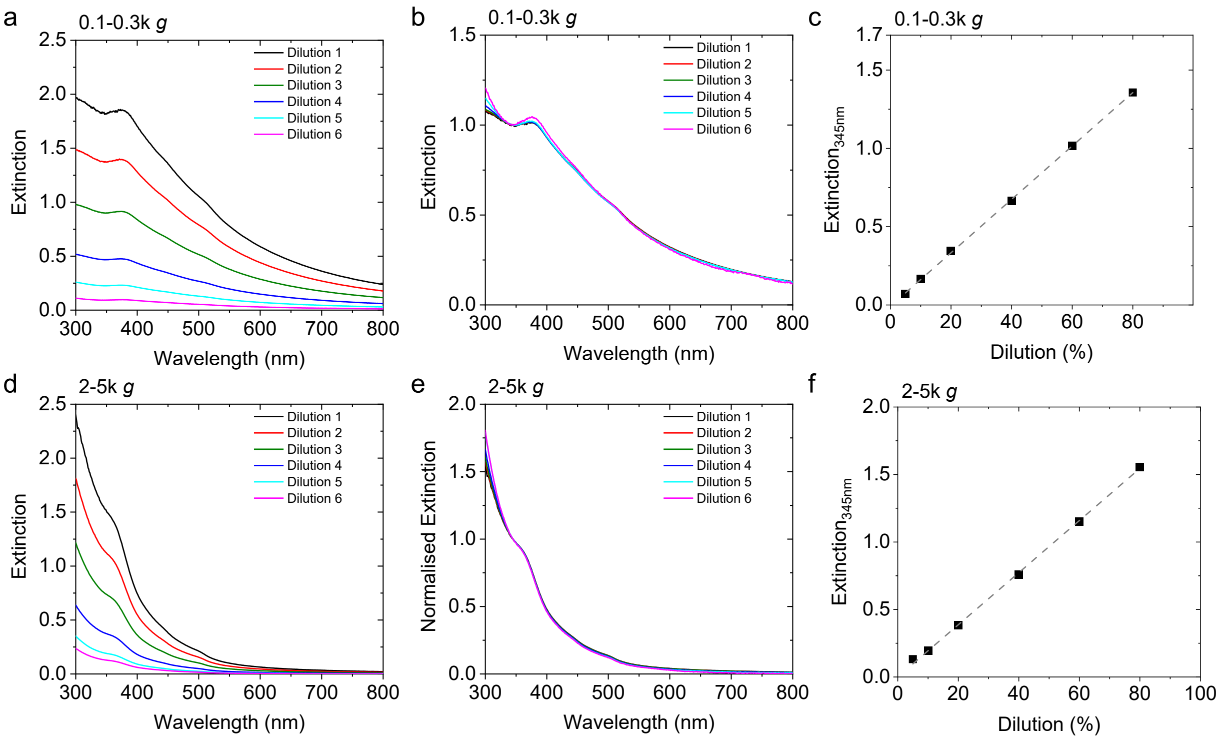

Figure S8: Dilution series of two size selected fractions. a-c) Sample 0.1-0.3k *g*, d-f) Sample 2-5k *g*. a,d) Extinction spectra measured with different dilutions, b, e) Normalized spectra confirming good overlap in the region of interest (~340-600 nm), c,f) Plot of extinction at 450 nm as function of dilution percentage of the parent dispersions.

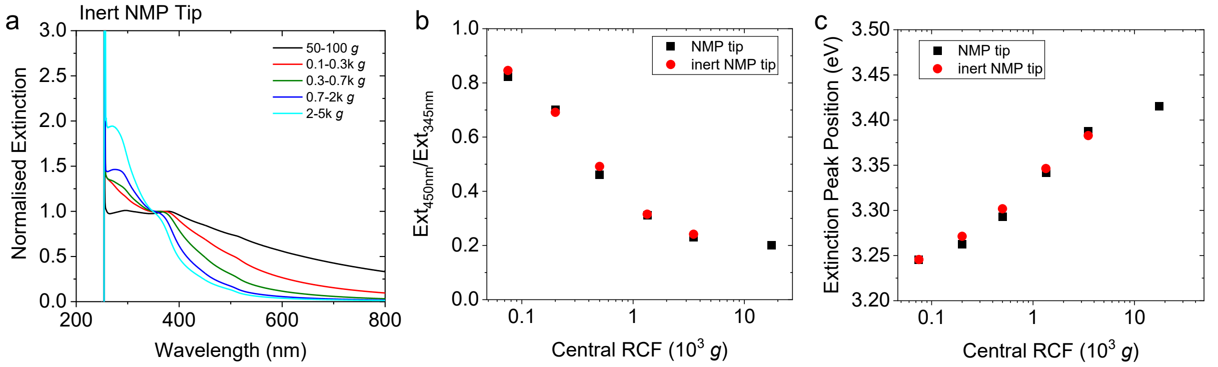

Figure S9: a) Normalised optical extinction after exfoliation using tip sonication in inert atmosphere (glovebox). b) Length metric extinction intensity ratio and c) thickness metric peak position plotted as function of central centrifugal acceleration comparing the exfoliation through tip sonication in NMP in ambience and under inert conditions.

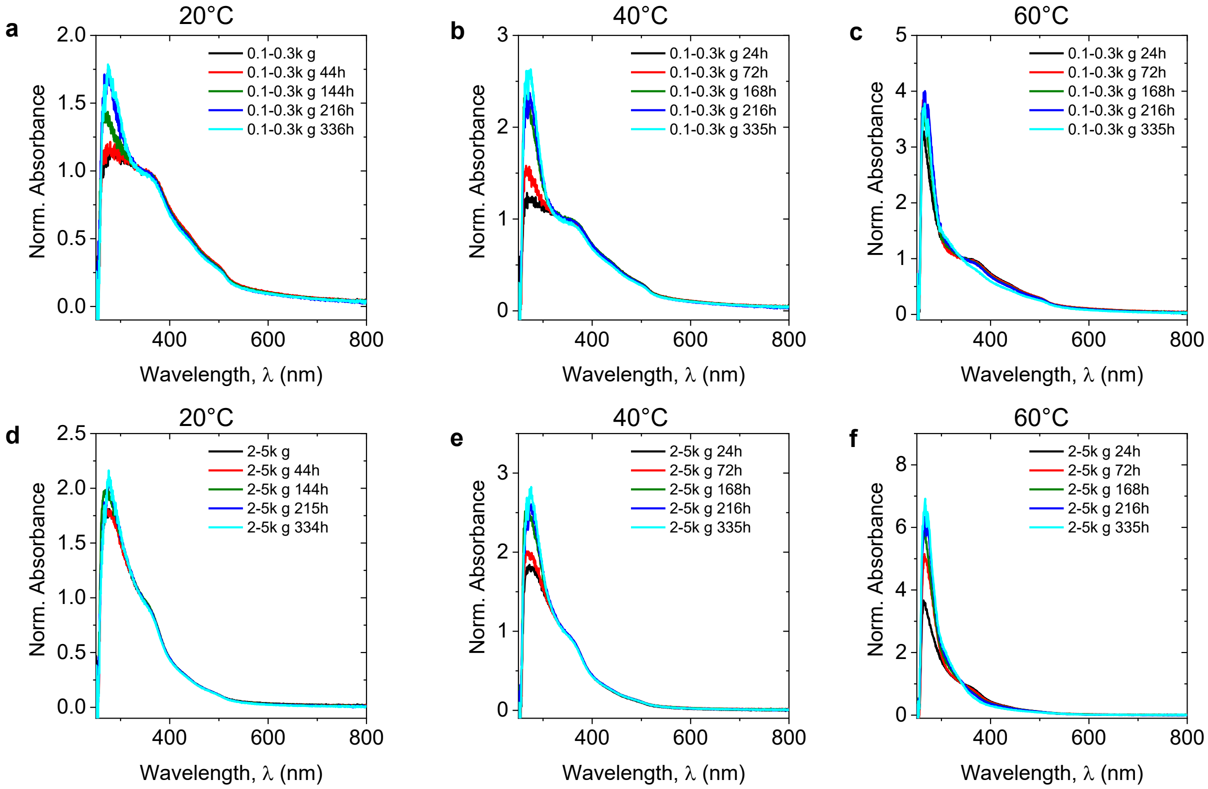

Figure S10: Normalised absorbance spectra measured at different time intervals after production and storage at different temperatures. Samples were left to equilibrate to room temperature prior to the measurement. A-C) Fraction 0.1-0.3 k g, D-F) 2-5k *g*.

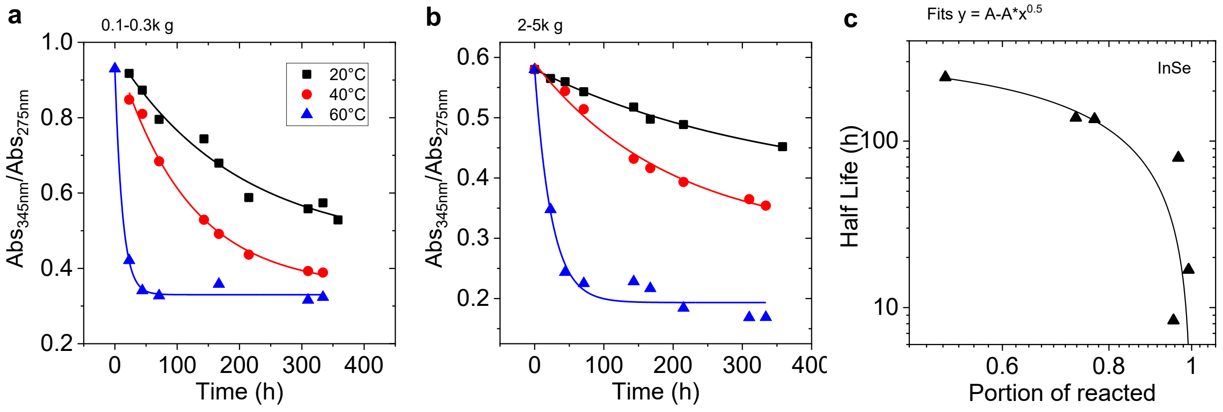

Figure S11: Fitted decay kinetics of the sample degradation. Intensity ratios of the absorbance at 345 nm to 275 nm plotted as function of time for a) Fraction 0.1-0.3k g, b) Fraction 2-5k g. Lines are exponential fits to extract half-life and portion of reacted material at infinite time. c) Half-life as function of portion of reacted material. The line is an empirical function that described the data.

3. Error estimation of spectroscopic metrics

Fitting the <*L*> metric data gives
$A = 0.001000 \pm 1.06416 \times 10^{-4}$
$B = 0.14858 \pm 0.00910$
$C = -7.19403 \times 10^{-4} \pm 1.12007 \times 10^{-4}$

With a covariance matrix of

	A	B	C
A	1.13E-08	-8.50E-07	1.17E-08
B	-8.50E-07	8.29E-05	-8.10E-07
C	1.17E-08	-8.10E-07	1.25E-08

Because standard errors of the measured datapoints are neglectable, the uncertainty in the estimated lateral size was obtained by propagation of the covariance matrix of the nonlinear least-squares fit. For

$$\mathrm{L(R)} = \frac{\mathrm{B - R}}{\mathrm{CR - A}},$$

the Jacobian is

$$\mathbf{J} = \left(\frac{\mathrm{L}}{\mathrm{CR - A}}, \frac{1}{\mathrm{CR - A}}, -\frac{\mathrm{RL}}{\mathrm{CR - A}}\right),$$

and the variance is calculated as

$$\sigma_{\mathrm{L}}^2 = \mathbf{J}^{\mathrm{T}}\Sigma\mathbf{J},$$

where Σ is the covariance matrix of the fitted parameters.

Fitting the layer-number calibration data gives

$y_0 = 3.24319 \pm 0.00626$
$A_1 = 0.44722 \pm 0.04667$
$t_1 = 5.10336 \pm 0.58532$

With a covariance matrix of

	y0	A1	t1
y0	3.92E-05	1.52E-04	-0.00286
A1	1.52E-04	0.00218	-0.02493
t1	-0.00286	-0.02493	0.34259

Because the standard errors of the measured peak positions are negligible, the uncertainty in the estimated layer number was obtained by propagation of the covariance matrix of the nonlinear least-squares fit. For

$$\langle N\rangle_{\mathrm{Vf}}(E_{ext}) = -t_1 \ln\left(\frac{E_{ext} - y_0}{A_1}\right),$$

the Jacobian is

$$\mathbf{J} = \left(\frac{t_1}{E_{ext} - y_0}, \frac{t_1}{A_1}, \frac{\langle N\rangle_{\mathrm{Vf}}}{t_1}\right),$$

and the variance is calculated as

$$\sigma_N^2 = \mathbf{J}^{\mathrm{T}}\mathbf{\Sigma}\mathbf{J},$$

where **Σ**is the covariance matrix of the fitted parameters.
